# pH-Responsive PVA-Based Nanofibers Containing GO Modified with Ag Nanoparticles: Physico-Chemical Characterization, Wound Dressing, and Drug Delivery

**DOI:** 10.3390/mi13111847

**Published:** 2022-10-28

**Authors:** Erfan Rahmani, Mehrab Pourmadadi, Nayereh Zandi, Abbas Rahdar, Francesco Baino

**Affiliations:** 1 School of Chemical Engineering, College of Engineering, University of Tehran, Tehran 11155-4563, Iran; 2 Department of Biomedical Engineering, University of Delaware, Newark, DE 19713, USA; 3 Protein Research Center, Shahid Beheshti University, Tehran 1983963113, GC, Iran; 4 Department of Medical Laboratory Science, School of Medicine, Qazvin University of Medical Sciences, Qazvin 34, Iran; 5 Department of Physics, Faculty of Science, University of Zabol, Zabol 98613-35856, Iran; 6 Institute of Materials Physics and Engineering, Department of Applied Science and Technology, Politecnico di Torino, 10129 Torino, Italy

**Keywords:** poly (vinyl alcohol), graphene oxide, silver nanoparticle, nanomaterials, curcumin, wound dressing, antibacterial effects, electrospinning

## Abstract

Site-specific drug delivery and carrying repairing agents for wound healing purposes can be achieved using the intertwined three-dimensional structure of nanofibers. This study aimed to optimize and fabricate poly (vinyl alcohol) (PVA)-graphene oxide (GO)-silver (Ag) nanofibers containing curcumin (CUR) using the electrospinning method for potential wound healing applications. Fourier Transform Infrared (FTIR) spectrophotometry, X-ray Diffraction (XRD), Scanning Electron Microscopy (SEM), Dynamic Light Scattering (DLS), and zeta potential were used to characterize the nanostructures. The mechanical properties of the nanostructures were subsequently examined by tensile strength and elongation test. As shown by MIC analysis of *E. coli* and *S. aureus* bacteria, the fabricated nanofibers had superior inhibitory effects on the bacteria growth. Ag nanoparticles incorporation into the nanofibers resulted in increased loading and encapsulation efficiencies from 21% to 56% and from 61% to 86%, respectively. CUR release from PVA/GO-Ag-CUR nanofiber at pH 7.4 was prevented, while the acidic microenvironment (pH 5.4) increased the release of CUR from PVA/GO-Ag-CUR nanofiber, corroborating the pH-sensitivity of the nanofibers. Using the in vitro wound healing test on NIH 3T3 fibroblast cells, we observed accelerated growth and proliferation of cells cultured on PVA/GO-Ag-CUR nanofibers.

## 1. Introduction

Human life is under threat from a variety of infections and the skin is of crucial importance in protecting people from dangerous pathogens such as bacteria and viruses. The major problem in wound healing is infection related to the secretion and aggregation of microorganisms in the wound area [1,2]. Surpassing this constraint may be as simple as wound treatment with antibiotics, which lowers the secretion of bacteria and microorganisms at the wound area [3,4]. Notwithstanding the many advantages of employing antibiotics for wound healing, some crucial drawbacks limit their widespread usage, including developing drug resistance, production of multidrug-resistant bacteria, and insufficient or slow response for the immune system to infections [5,6]. These faults may be remedied by substitutive treatments, such as targeted nanotechnology-based approaches, decreasing the administration of widely used antibiotics in the wound healing process [7]. Wound dressing platforms have become popular among different wound healing approaches for biocompatibility, physical and chemical stability, porous structure, flexibility, and tissue-adhesive and mechanical properties [1,8,9,10]. A suitable wound dressing may maintain the acceptable level of moisture and temperature around wound environment, create a barrier against penetration of various microorganisms and infection, allow adequate flow of oxygen, and enable exudate absorption [11,12]. Hence, placing a suitable wound dressing platform on the damaged area allows for an accelerated wound healing process. An ideal wound dressing should fulfil several requirements for facilitating wound healing, including good mechanical flexibility under tension [13], physical and chemical stability [14], ease of application and removal [15], biocompatibility [16], and prevention of wound infection [17]. Regarding their biocompatibility, chemical stability, and high porosity, polymers have been extensively incorporated into biomedical platforms [18]. Synthetic polymers are a favorable option for wound dressing because of their low toxicity and biodegradability, failing to cause harm to the living tissues upon their enzymatic degradation [19]. Among many other methods, electrospinning is considered as a cost-efficient, simple, and efficient method for wound dressing. In fact, promising properties associated to polymeric nanofibers make electrospinning a perfect technique for the fabrication of drug-loaded electrospun nanofibers. Furthermore, it was stated that nanofibers are a useful platform for releasing drugs in a controllable manner in the wounded area and reducing the adverse side effects of drugs [13,17,20].

Poly (vinyl alcohol) (PVA) belongs to the class of water-soluble biocompatible synthetic polymers. Regarding its facile synthesis and high purity, PVA has received special attention among researchers [1,13]. Based on its unique properties, including a high degree of polymerization of 200–3000 [21], elastic nature [22], high degree of swelling [23] and chemical stability [24], PVA can be a suitable option for wound dressing and biomedical applications. These properties make PVA suitable for many medical applications such as the regeneration of bone fractures [25], dentistry [26], skin tissue repair [27], artificial blood [28] and cornea vessels [29]. Its ability to keep a moist wound environment and capability of absorbing exudates from the wound surface make PVA one of the most common synthetic polymers used in wound dressing [30]. Furthermore, thanks to its non-adhesive property, PVA offers a unique advantage for easily separating the dressing from the wound [31]. For instance, PVA-based wound dressing was recently reported by Kokabi et al. [32], who fabricated PVA–clay hydrogel nanocomposites by the freeze-thaw method. They concluded that PVA plays a crucial role in keeping a moist environment and significantly absorbing fluid, thus accelerating the wound healing process. While it has certain drawbacks, including a lack of biological activities and poor mechanical properties, PVA is a promising candidate for wound dressing in light of the characteristics mentioned above.

Graphene oxide (GO) is a promising two-dimensional carbon material for tissue engineering applications due to its unique characteristics, i.e., high surface area, presence of abundant oxygen functional groups (such as hydroxyl (C-OH), epoxide (C-O-C), carbonyl (C=O) and carboxyl (-COOH) groups), and structural and electrical properties [33,34]. The presence of oxygen functional groups provides GO sheets with good dispersity in water for forming stable colloidal suspension [35]. GO has been recently applied as structural support to anchor gold nanoparticles on it for catalytic applications [36]. It was found that GO may be used as an antibacterial agent by physical damage to the cell membrane [37]. Additionally, the multifunctional imaging and cancer-sensing applications of GO composites have been reported in recent years [38,39]. Esmaeili et al. [40] created a GO-based biosensor using fluorescence and the optical properties of GO and observed the emission of the fabricated biosensor in three main fluorescence regions.

Inorganic nanomaterials have attracted substantial attention in antibacterial applications regarding their large surface area-to-volume ratio and controllable size and shape morphology [41]. Blending metal and metal oxide nanoparticles, including silver (Ag), titanium oxide (TiO_2_), copper (Cu), zinc oxide (ZnO), and copper oxide (CuO), has caused a notable improvement in the antibacterial effect of these nanomaterials. Due to its good antibacterial properties, acceptable mechanical and thermal stability, stimulatory effect on cell proliferation and angiogenesis, and activation of cytokines and growth factor mechanisms, Ag nanoparticles are highly attractive in the field of wound healing [42]. Notwithstanding the many advantages of employing Ag for wound dressing, the drawback related to its concentration limits its widespread employment [43]. For instance, significant toxicity was detected in rainbow trout gill fish cell line RT-W1 cultured with nanoparticles containing 25 mg/L of silver [44]. This fault may be remedied by lower Ag concentrations, making Ag more useful in wound dressing.

Curcumin (CUR) belongs to the flavonoid group of polyphenols [45] and has been used as a traditional therapeutic substance in medicine since ancient times [46]. Based on its ability to reduce the expression of cyclin D1 and Bcl-2, activate caspase, and stimulate p53 expression, CUR can be used for apoptosis induction in cancer cells [47]. The efficacy of CUR on breast cancer cells was reported in previous studies [48,49,50]. In fact, CUR acts as an antitumor agent by a p53-dependent pathway on MCF-7 cells [51]. The abovementioned attributes of CUR make it suitable for cancer treatment, although it still has some limitations including low permeation, poor solubility, and a shorter half-life, which can compromise the efficacy of CUR in cancer therapy [52,53].

In this study, we prepared GO using the Hummer’s method and then synthesized GO-Ag-CUR nanostructures prior to the electrospinning with PVA for fabricating nanofibrous wound dressing nanocomposite platforms. In regard to finding the most suitable processing conditions for the synthesis of GO-Ag-CUR nanostructures with optimum swelling behavior, we opted for the Response Surface Methodology (RSM). To investigate the pH-responsive release of CUR as the model drug from the fabricated nanofibers, the in vitro release study was performed. Moreover, our research aimed to study the effects of the fabricated nanofibers on regenerating wounds using NIH 3T3 fibroblasts. To the best of our knowledge, this is the first original study that benefits from the simultaneous use of PVA, GO, Ag, and CUR for studying their antibacterial activities and mechanical attributes.

## 2. Materials and Methods

### 2.1. Materials

Sigma (USA) provided fetal bovine serum (FBS), dimethylsulfoxide (DMSO), Dulbecco’s Modified Eagle’s Medium (DMEM) High Glucose, and 3-(4,5-dimethylthiazol-2-yl)-2,5-diphenyl-tetrazolium bromide (MTT). The American Type Culture Collection (ATCC) provided NIH 3T3 fibroblasts cell line. Penicillin/streptomycin and 0.25% (*w*/*v*) trypsin-0.1% (*w*/*v*) ethylenediaminetetraacetic acid (EDTA) was purchased from Solarbio (Beijing Solarbio Science and Technology, China).

### 2.2. GO Fabrication

GO was prepared through Hummer’s method according to the previously reported literature [54]. Firstly, 1 g of diammonium hydrogen citrate was dispersed in 20 mL of sulfuric acid 98%. Then, 3 g of potassium permanganate (KMnO_4_) was slowly introduced to the solution. Next, 50 mL distilled water was gradually introduced over 30 min to the solution in a drop-by-drop manner. After 10 min, 100 mL distilled water and 35 mL hydrogen peroxide (H_2_O_2_) were added into the solution, and the final solution was stirred (~500 rpm) for 24 h.

### 2.3. GO-Ag and GO-Ag-CUR Fabrication

To begin, at room temperature with magnetic stirring (300 rpm) to create 75 g GO, 1 g of sodium borohydride (NaBH_4_) was slowly added to the prepared solution. After centrifugation (10,000 rpm, 10 min), the mixture was washed with ethanol to remove unreacted components. After adding 15 mL deionized water to the synthesized GO, the resulting solution was sonicated for about 10 min using a probe sonication and ultrasound to obtain a homogenous solution. This stage included the addition of silver nitrate (AgNO_3_) to the GO solution to create a 7 mg/L GO-Ag nanohybrid through dissociation of AgNO_3_ to Ag^+^ cation and nitrate anion (NO_3_^−^) [55]. For synthesizing the GO-Ag-CUR nanostructure with a concentration of 7.5 mg/L, 30 mg of CUR was added to the prepared nanohybrid of GO-Ag under vigorous stirring conditions (~600 rpm for 10 min). In the last step, the produced nanostructure was left in liquid nitrogen for 5 min before transferring into the freeze dryer.

### 2.4. PVA/GO-Ag-CUR Nanofibers Fabrication

Firstly, 50 mg of prepared GO-Ag-CUR was slowly added into 5 mL of deionized water acid, making use of a magnetic stirrer (400 rpm) to reach a homogeneous solution. Then, 0.3 g of PVA was dispersed in 3 mL deionized water under vigorous stirring condition (~500 rpm) to reach a homogenous solution of 0.1% (*w*/*v*) before adding to the prepared solution. In this stage, the prepared GO-Ag-CUR solution was quickly injected into the PVA hydrogel at room temperature and stirred (~300 rpm) for 2 h. For being injected, the homogenous mixture was placed into a 5 mL syringe, with a 22-gauge blunt end needle. Voltage, injection flow rate, and gap distance were adjusted to 15 kW, 0.2 mL/h, 15 cm, respectively. In order to assure that the evaporation of solvents is completed, nanofibers were left standing for one day. In order for strong crosslinking to take place, the prepared nanofibers were placed in a desiccator with glutaraldehyde (GA) vapor for 24 h at 50 °C. Finally, nanofibers were washed with phosphate-buffered solution (PBS) and dried overnight in a laminar flow hood to remove unreacted GA.

### 2.5. Characterization

To determine interactions between components in the samples of GO, GO-Ag, GO-Ag-CUR, and PVA/GO-Ag-CUR, Fourier Transform Infrared (FTIR, Thermo AVATAR, Chicago, IL, USA) spectrophotometry and X-ray diffraction (XRD, PHILIPS, PW1730, Amsterdam, Netherlands) were used. Zeta potential analysis was performed to investigate the surface charge of nanostructures. Dynamic Light Scattering (DLS) was used to evaluate the size distribution. Scanning Electron Microscopy (SEM) was also employed to characterize PVA/GO-Ag-CUR morphology. Field-Emission Scanning Electron Microscopy (FESEM, TESCAN MIRA III, Brno, Czechia) was also used to characterize the GO-Ag-CUR morphology. Mechanical properties were investigated by tensile tests (testing machine STM-20).

### 2.6. Optimization

A three-level-two-factor central composite design (CCD) approach-based response surface methodology (RSM) was employed in order to investigate and optimize the impacts of concentration of GO-Ag-CUR nanostructure and volume ratio of GA to GO-Ag-CUR nanostructure on the absorbed water of PVA/GO-Ag-CUR nanofiber. Levels of independent variables and their experimental ranges are presented in Table 1. Dry weighted PVA/GO-Ag-CUR nanofibers (W_ij_) was added to 10 mL PBS at pH 7.7 at 37 °C. The nanofibers were collected from falcons after 24 h, and excess water was filtered through filter paper. Finally, the nanofibers were weighed using a digital scale (W_if_). The water absorption of the nanofibers was calculated using Equation (1).

(1) $$\mathrm{Water}\;\mathrm{absorption}\;(\%)=\frac{\left(W_{\mathrm{if}}-W_{\mathrm{ij}}\right)}{\left(W_{\mathrm{ij}}\right)}\times 100$$

### 2.7. Biodegradability

A biodegradability test was employed to study the biodegradability behavior of the optimized PVA/GO-Ag-CUR nanofiber. Firstly, 1 cm × 1 cm dry sample was weighed (W_1_) prior to being submerged in 10 mL of PBS solution at pH 7.4. Then, the sample incubated for specific time intervals of 7, 14, 28, 42, 56, 72 and 96 h at 37 °C. At adopted time intervals, the sample was extracted and weighed (W_2_). The percentage of mass loss from PVA/GO-Ag-CUR was determined employing the Equation (2):

(2) $$\mathrm{Degradation}\;(\%)=\frac{\left(W_{1}-W_{2}\right)}{\left(W_{1}\right)}\times 100$$

### 2.8. Antibacterial Study

Minimum Inhibitory Concentration (MIC) assay was used to study the antibacterial activity of GO, GO-Ag, and GO-Ag-CUR nanostructure against *E. coli* and *S. aureus* bacteria. Mueller Hinton broth media were used for the incubation of the bacteria for 24 h at 37 °C. Next, 0.5 McFarland solution was obtained by adding the physiological serum to cultured bacteria with volume proportion of 0.01. Each of the wells was little by little added with 100 µL of sterilized growth medium after fixing the optical density (OD_600_) at 0.11, and then all wells excluding the last one were charged with 5 µL of bacteria suspensions. After adding 100 µL of the sterilized GO sample to the first well, an equal amount (100 µL) was extracted from the first well and added to the second well. The same action was carried out for other wells until well column 10 was reached. Well column 10 holding bacteria and culture medium was regarded as the positive group, and well column 12 containing culture medium was regarded as the negative group. The same method was repeated for GO-Ag and GO-Ag-CUR samples. Ultimately, after the incubation of all plates at 37 °C for 24 h, MIC for each row were calculated employing ELISA Reader (BioTek EIA reader, Santa Clara, CA, United States).

### 2.9. CUR Encapsulation and Loading Efficiency Measurement

Next, 1 mg of lyophilized nanoparticles, GO-Ag-CUR, were carefully weighed and dispersed in 1 mL of PBS before the addition of ethyl acetate. When the solution was shaken, the ethyl acetate phase was separated. A UV–Vis spectrophotometer (UV-T60U; PG Instrument, Lutterworth, England) at 419 nm has been employed to measure the quantity of unbound CUR in ethyl acetate extract [56]. Encapsulation and loading efficiencies of CUR were measured employing Equations (3) and (4) [57].

(3) $$\mathrm{Encapsulation}\;\mathrm{efficiency}\;(\%)=\frac{\left(\mathrm{Entire}\;\mathrm{quantity}\;\mathrm{of}\;\mathrm{CUR}\right)-\left(\mathrm{Unbound}\;\mathrm{quantity}\;\mathrm{of}\;\mathrm{CUR}\right)}{\left(\mathrm{Entire}\;\mathrm{quantity}\;\mathrm{of}\;\mathrm{CUR}\right)}$$

(4) $$\mathrm{Loading}\;\mathrm{efficiency}\;(\%)=\frac{\left(\mathrm{Entire}\;\mathrm{quantity}\;\mathrm{of}\;\mathrm{CUR}\right)-\left(\mathrm{Unbound}\;\mathrm{quantity}\;\mathrm{of}\;\mathrm{CUR}\right)}{\left(\mathrm{Entire}\;\mathrm{quantity}\;\mathrm{of}\;\mathrm{Nanostructure}\right)}$$

### 2.10. In Vitro Drug Delivery

For the purpose of comparing and investigating the release of CUR from PVA/GO-Ag-CUR nanofiber at pH = 5.4 and pH = 7.4, a dialysis technique was employed to identify the release pattern of CUR from the fabricated nanofiber. For the next 96 h, Spectrum-Labs dialysis bags (cut off Mw = 10–12 kDa) with 5 mL of nanofiber mixture containing the drug were submerged in 30 mL of two separate phosphate buffers with 20% *v*/*v* ethanol at 37 °C [58]. PBS (NaCl 0.138 M; KCl 0.0027 M) has been used for pH = 7.4 to dilute the lyophilized aptamer powders, which were then adjusted to pH = 5.4 using a pH meter and HCl solution. The release medium was regularly removed in order to calculate the release of CUR within buffers. A new equal volume of fresh buffers was put back to maintain a steady volume. Using a UV Vis spectrophotometer (UV-T60U; PG Instrument, Lutterworth, England) to measure absorption, the samples were evaluated spectrophotometrically at a 419 nm [56]. To estimate the percent of the discharged drug, Equation (5) can be used:

(5) $$\mathrm{CUR}\;\mathrm{released}\;(\%)=\frac{\left[\mathrm{CUR}\right]\mathrm{rel}}{\left[\mathrm{CUR}\right]\mathrm{load}}*100$$

where [CUR]load denotes the amount of CUR entrapped throughout the nanofiber, and [CUR]rel is the amount of CUR discharged from the nanofibers.

### 2.11. Kinetics of Release

The in vitro results for the release process were matched to models (zero-order, first-order, Korsmeyer–Peppas, and Higuchi models) to investigate the procedure of CUR delivery from the nanostructure at pH 7.4 and 5.4. It is possible to identify the best fitting model by looking at the correlation coefficient (R^2^).

- Zero-order model

The zero-order model would fit the release kinetics exhibiting consistent pharmaceutical concentrations throughout the release [59]. The release of zero-order model may be described as follows:

Q_t_ = Q_0_ + k_0_ t (6)

where Q_t_ represents the quantity of drug released at time t, Q_0_ is the drug beginning quantity, and k_0_ is the zero-order release constant.

- First-order model

When simulating the release of water-soluble medicines from porous materials, the first-order model is a semi-empirical kinetic model that is often employed [60,61]. The release of the first-order model may be described as follows:

Ln Q_t_ = Ln Q_0_ + k_1_ t (7)

where Q_t_ denotes the quantity of drug released at time t, Q_0_ represents the drug beginning quantity, and k_1_ is the constant of the first-order model. We also tested this model for the sake of completeness but we cannot ignore that CUR is poorly soluble in water; thus, we guess that this model will be not very suitable in this case.

- Korsmeyer–Peppas model

The Korsmeyer–Peppas approach is a simple yet thorough quasi-empirical formula that aids in the modeling of unidentified release mechanisms [60,62]. The release of Korsmeyer–Peppas model may be described as follows:

M_t_/M_∞_ = k t^n^ (8)

where M_t_/M_∞_ represents the percentage of released drug at time t, k is the release constant of the Korsmeyer–Peppas model, and n denotes the release exponent. If n ≤ 0.5, Fickian diffusion is the primary release method, and the solvent infiltration determines the rate of release. Anomalous transport is the primary method of release if 0.5 < n < 1, and the amount of release is governed by both Fickian and relaxing processes depending on state transitions and swellings in polymers expanding in the solvent [63,64].

- Higuchi model

Analysis of release from water-soluble and poor soluble matrix structures is carried out using the Higuchi model [65]. The release of the Higuchi model may be described as follows:

M_t_/M_∞_ = k_h_ t^0.5^ (9)

where M_t_/M_∞_ implies the released proportion of medicine at time t, k_h_ is the release constant of Higuchi.

### 2.12. Cytotoxicity Analysis

MTT assays have been used to determine the toxicity of GO-Ag, GO-Ag-CUR, and PVA/GO-Ag-CUR on NIH 3T3 fibroblast cell line. Based on former studies [66], after adding the NIH 3T3 fibroblast cell line into a 24-well cell culture plate, the cells were given 24 h attachment period in the incubator at 37 °C and 5% CO_2_. The next step was to introduce the nanostructures with MIC concentration to each well and inside the incubator over 24 h at 37 °C. The cells were cultured as control without any treatment in DMEM basic medium including 10% FBS and 1% penicillin/streptomycin for 24 h. After 24 h, 50 μL of 5 mg/mL MTT solution was added to each well with the plates incubated for 4 h at 37 °C. To dissolve the formazan crystals, the wells were rinsed with PBS buffer and then filled with 150 µL of DMSO and stirred vigorously (20 min). Using an ELISA reader with the aim of measuring optical density, the wells were read at 570 nm. For studying cell adhesion and morphology via SEM images, cells were fixed on the nanofibers through the following steps. In the first step, the nanofibers were taken out of the incubator, each well culture medium was discharged, and 200 μL of GA was added to them. In the second step, GA was allowed to be in contact with the nanofibers for 3 h and after that was moved away from the wells and washed with PBS. In the final step, wells were charged with 200 μL of graded ethanol solution (50, 75, and 96%) for 10 min. Finally, the nanofibers were taken out of the wells and dried at ambient temperature and photographed with SEM for cell adhesion assay.

## 3. Results

### 3.1. Characterization of GO, GO-Ag and GO-Ag-CUR Nanostructures

#### 3.1.1. FTIR

**Figure 1:**
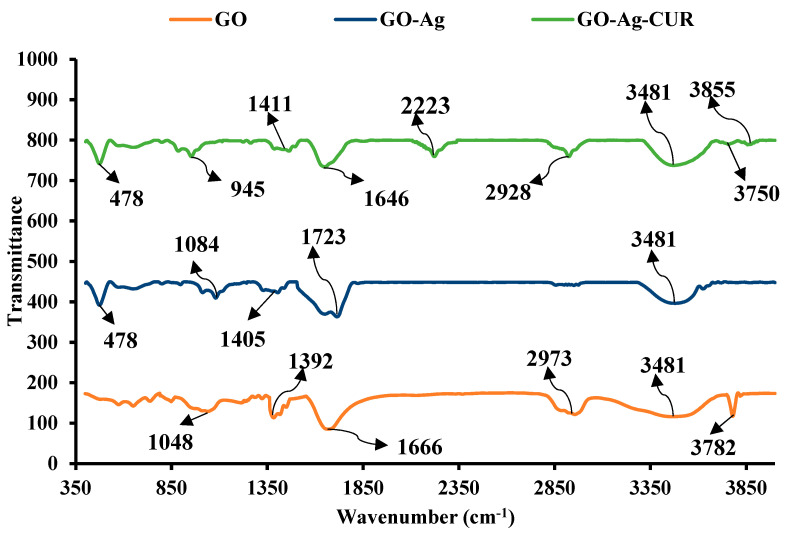
The FTIR spectra of GO, GO-Ag, GO-Ag-CUR.

FTIR analysis was conducted in order to identify chemical bonds between GO, Ag, and CUR. FTIR spectra for GO, GO-Ag, and GO-Ag-CUR are reported in Figure 1. The 3872 cm^−1^ and 3481 cm^−1^ bands in the FTIR spectrum of GO might be attributed to the O-H stretching present in hydroxyl and carboxyl groups. At 2973 cm^−1^, the C-H stretching may be identified. The absorption band at around 1666 can be ascribed to the C=O stretching oscillation of carboxyl group. The band observed at 1392 cm^−1^ is possibly due to C-OH stretching. GO exhibits bending vibration band at 1048 attributable to the C-O stretching of the epoxy and the alkoxy groups. All of the results are in accordance with prior investigations on the FTIR spectrum of GO [67].

The FTIR spectrum of GO-Ag were examined to validate the inclusion of Ag nanoparticles. The band at 478 cm^−1^ proved the presence of Ag in the GO-Ag nanostructure [68]. The intensity of the band at 1048 cm^−1^ ascribed to the C-O stretching of the epoxy and the alkoxy groups decreased, and its position was shifted towards a higher frequency (1084 cm^−1^). Because of the interactions in molecular level between GO and Ag, the bands at 1392 and 1666 cm^−1^ in GO were displaced to 1405 and 1723 cm^−1^, respectively. Furthermore, the band at 2973 cm^−1^ ascribed to C-H stretching disappeared in the FTIR spectrum of GO-Ag. The interaction between GO and Ag nanoparticles was validated by the shift in the FTIR range, appearance and disappearance of bands, and decrease in intensity that occurred whenever Ag was mixed with the GO nanostructure.

In the FTIR spectrum of GO-Ag-CUR, all distinguishing bands of GO were observed. The bands at 3855, 3750, and 3481 cm^−1^ in the spectrum of GO-Ag-CUR are linked to the O-H stretching in hydroxyl and carboxyl groups. The bands at 2928 cm^−1^ detected in the spectrum of GO-Ag-CUR signified the C-H stretching of CUR, also detected in the FTIR spectrum of GO. Moreover, the presence of the bands at 1646 and 1411 cm^−1^ may have been due to C=O stretching oscillation of CUR. It has been determined that the band at 2223 cm^−1^ is due to carbon-carbon triple bond (–C≡C–). The band at 945 cm^−1^ can be ascribed to C-O-C stretching oscillation. The band at 478 cm^−1^ might be responsible for the presence of Ag [68]. When the components and drug have electrostatic interactions, the band intensity proportion at 2950 cm^−1^ is lowered, which shows the enhancing the pharmaceutical conjugation with the nanostructure. The band at 3454 cm^−1^ is attributable to N-H and O-H stretching exhibited greater intensity in the FTIR spectrum of GO-Ag-CUR, as compared to FTIR spectrum of GO-Ag. Because of the electrostatic associations between the protonated amine groups of CUR and the constituents of the fabricated nanostructure, the effective loading of CUR into the GO-Ag-CUR nanostructure was proved [69].

#### 3.1.2. XRD

**Figure 2:**
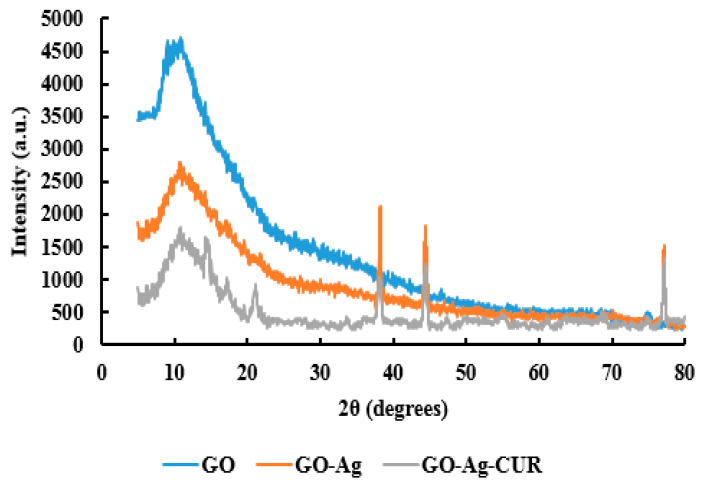
The XRD patterns of GO, GO-Ag, and GO-Ag-CUR.

XRD was performed to further confirm the nanostructure formation. The XRD patterns of GO, GO-Ag, and GO-Ag-CUR are presented in Figure 2. A broad halo appeared in the GO spectrum at 2θ of 11.5°, corresponding to the (001) plane [70]. The wide peak at 11.5° was also present in the XRD spectrum of GO-Ag, corroborating the presence of GO nanoparticles in GO-Ag nanostructure. The bands at 2θ = 38.1, 44.4, and 77.2° in the pattern of GO-Ag nanostructure can be ascribed to the (111), (200), and (311) planes of the face centered cubic (FCC) structure of the Ag nanoparticles, respectively, confirming the presence of Ag nanoparticles in the GO-Ag nanostructure [71]. The XRD of the GO-Ag-CUR nanostructure showed new peaks at 2θ = 14.6, 17.2, and 21.1° compared to the XRD pattern of GO-Ag, showing the successful load of the drug in the nanostructure and the crystalline state of the drug [72].

#### 3.1.3. DLS

**Figure 3:**
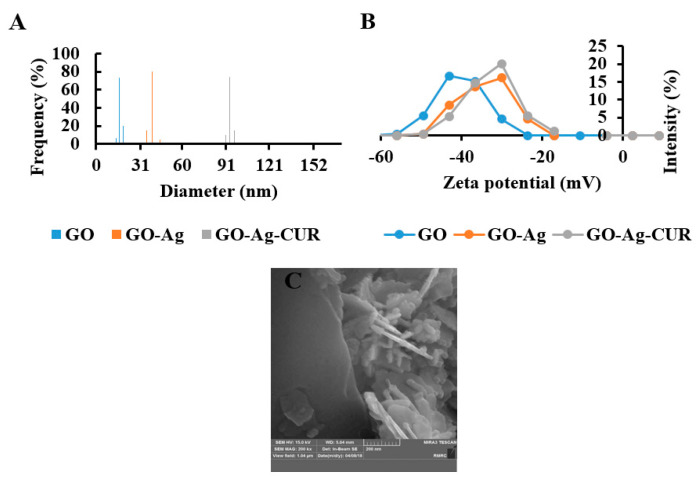
(**A**) DLS spectra of GO, GO-Ag, and GO-Ag-CUR (**B**) Zeta potential of GO, GO-Ag, and GO-Ag-CUR (**C**) FESEM image of GO-Ag-CUR.

The size distribution profile and polydispersity of GO, GO-Ag, and GO-Ag-CUR nanostructures were studied using DLS analysis (Figure 3A). The average GO nanoparticle size was reported to be 16.2 ± 3.8 nm. The average GO-Ag and GO-Ag-CUR nanostructures’ size were reported to be 37.2 ± 6.4 nm and 97.6 ± 1.4 nm, respectively. These increases in average sizes strongly suggest the incorporation of Ag nanoparticle and CUR into the GO layers. Furthermore, the DLS result of all samples showed a peak intensity of nearly 100%, which represents monodisperse distribution. The term monodispersity herein refers to the uniform nanostructures in both size and shape.

#### 3.1.4. Zeta Potential

In order to study the chemical stability of GO, GO-Ag, and GO-Ag-CUR nanostructures, zeta potential measurement was performed (Figure 3B). Values of zeta potential > +30 mV or <−30 mV may be considered stable. Zeta potential obtained from all groups was less than −30 mV, corroborating the nanostructures’ stability. The zeta potential of GO was higher than GO-Ag and GO-Ag-CUR nanostructures. This finding confirms the intermolecular interactions between components in GO-Ag and GO-Ag-CUR nanostructures, resulting the neutralization of charge in GO-Ag and GO-Ag-CUR nanostructures compared to GO.

#### 3.1.5. SEM

The morphological analysis of GO-Ag-CUR nanostructure is displayed in Figure 3C. The nanostructure has a layered structure, indicating the successful synthesis of GO. Furthermore, Ag and CUR are distributed homogeneously within the GO sheets. There is a satisfactory agreement between our SEM image and reported morphology of bacterial cellulose/GO-Ag nanocomposites for wound dressing by Mohammadnejad et al. [8]. Mitra et al. [73] developed CUR-loaded GO-based scaffold for wound healing applications: the layered structure of synthesized GO and entrapped CUR within GO layers reported in that study is consistent with our morphological analysis.

### 3.2. Characterization of the PVA/GO-Ag-CUR Nanofibers

#### 3.2.1. FTIR

**Figure 4:**
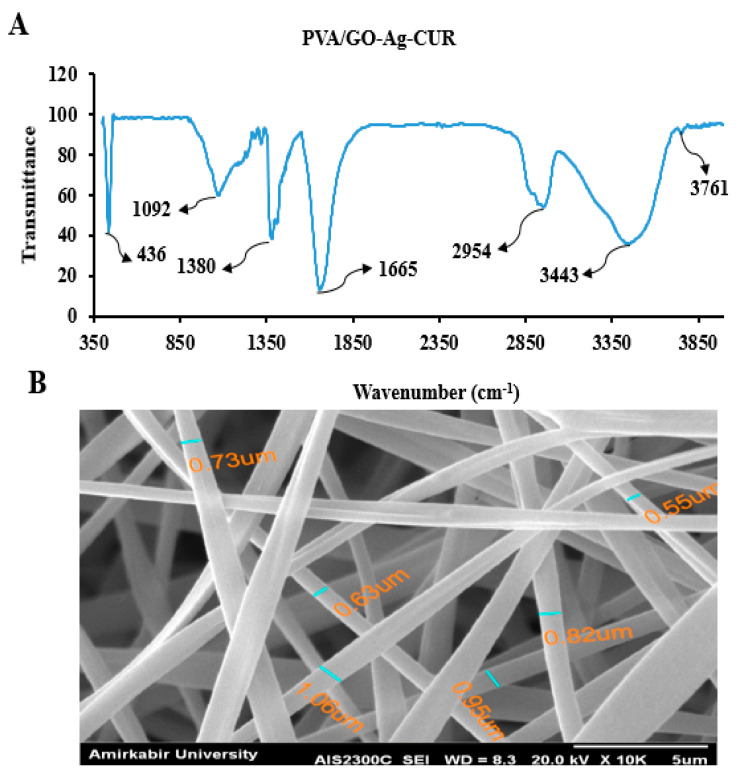
(**A**) FTIR spectrum of PVA/GO-Ag-CUR (**B**) SEM image of PVA/GO-Ag-CUR nanofibers.

The FTIR spectrum of PVA/GO-Ag-CUR were examined to validate the inclusion of the PVA (Figure 4A). The FTIR spectrum of PVA/GO-Ag-CUR showed characteristic bands of PVA in the 1665 cm^−1^ (C=O), 2954 cm^−1^(C-H), and 3443 cm^−1^ (O-H) ranges. The band at 3761 cm^−1^ is linked to the O-H stretching vibration of hydroxyl and carboxyl groups. The bands at 1380 and 1092 cm^−1^ might be due to carboxyl group’s C=O stretching and ketone group’s C-O stretching, respectively. The band at 436 cm^−1^ proved the presence of Ag in PVA/GO-Ag-CUR nanostructure.

#### 3.2.2. SEM

The morphology of PVA/GO-Ag-CUR nanofibers was illustrated by the SEM image in Figure 4B. According to Figure 4B, the nanofibers have a porous 3-D structure with an average diameter of 0.79 μm. Furthermore, a smooth surface and nearly similar diameter are visible in the electrospun fibers.

### 3.3. Optimization of the Swelling Behavior

For the purpose of studying and optimizing the water-uptake ability of PVA/GO-Ag-CUR, the CCD consisting of three levels was employed. Concentration of GO-Ag-CUR (A) (13, 20, and 27 mg/mL) and volume ratio of GA to GO-Ag-CUR (B) (2, 3.5, and 5 *v*/*v*) were selected as effective factors. The significant factors, curvature, and their interaction effects on response were calculated using Minitab software. Employing a regression analysis on the obtained experimental results (Table 1), the relation between the water-uptake ability of PVA/GO-Ag-CUR and the test parameter was represented based on the second-order polynomial equation. The analysis of variance results for the CCD is listed in Table 2. To interpret whether each of parameters and their interaction are significant to be consider or not, F-value and *p*-value were used. Values of *p* < 0.05 suggested that the terms are significant. All the parameters were non-significant. Next, the parameter with the highest *p*-values (AB) was not considered, and the analysis was repeated again. According to *p*-values, all parameters have a *p*-value less than 0.05 (Table 3). To establish a regression model, the following second-order polynomial equation was fitted to the experimental responses and test variables:

Water-uptake ability (%) = 140.53 − (4.79 × A) − (26.70 × B) + (0.14 × A^2^) + (2.59 × B^2^) (10)

**Figure 5:**
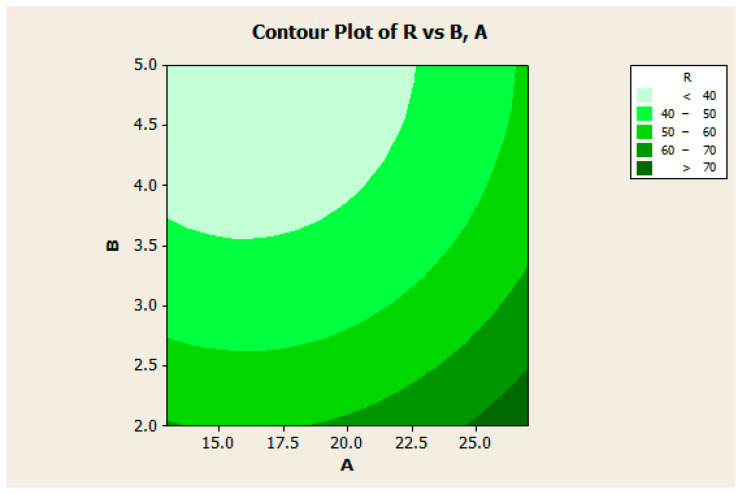
Contour plot for interaction between concentration of GO-Ag-CUR and volume ratio of GA to GO-Ag-CUR.

Figure 5 indicates contour plot between the combined effect of concentration of GO-Ag-CUR and volume ratio of GA to GO-Ag-CUR on the water-uptake ability (%). The figure shows that the water-uptake ability (%) increases with a decrease in the volume ratio of GA to GO-Ag-CUR. On the other hand, water-uptake ability (%) decreases with a decrease in the concentration of GO-Ag-CUR nanostructure. Considering these findings, we can conclude that since the significant density of cross-links causes the nanostructure matrix to be compact with limited space between components, the swelling potential of the nanostructure diminishes with increasing GA as a cross-linker. The optimized water-uptake ability (77.11%) occurred at the GO-Ag-CUR concentration of 27 mg/mL and GA/GO-Ag-CUR volume ratio of 2.

Figure 5 shows that an increase or decrease in the A factor (concentration of GO-Ag-CUR) causes a much slighter change in water-uptake ability compared to B (volume ratio of GA to GO-Ag-CUR), suggesting the meaningful effect of concentration of GO-Ag-CUR on water-uptake ability.

### 3.4. Biodegradability

**Figure 6:**
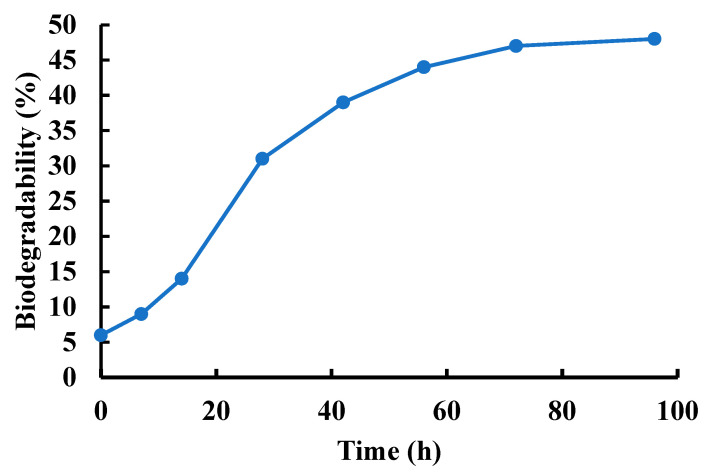
The degradation rate of optimized PVA/GO-Ag-CUR nanofiber in pH 7.4 at 37 °C.

We studied the in vitro degradation of optimized PVA/GO-Ag-CUR nanofiber in pH 7.4 at 37 °C. Figure 6 displays the nanofiber’s degradation curve over 96 h. The degradation profile was characterized by an early rapid degradation in the first 28 h succeeded by a sluggish degradation. As Figure 6 depicts, the degradation of PVA/GO-Ag-CUR nanofiber was 42% within 96 h, whereas 22% of PVA/GO-Ag-CUR nanofiber was degraded within 21 h. In research carried out by Bahadoran and co-workers [74], a maximum degradation of about 50% for PVA-sodium alginate hydrogel-based scaffold was reported after passing 4 weeks.

### 3.5. Mechanical Properties

**Figure 7:**
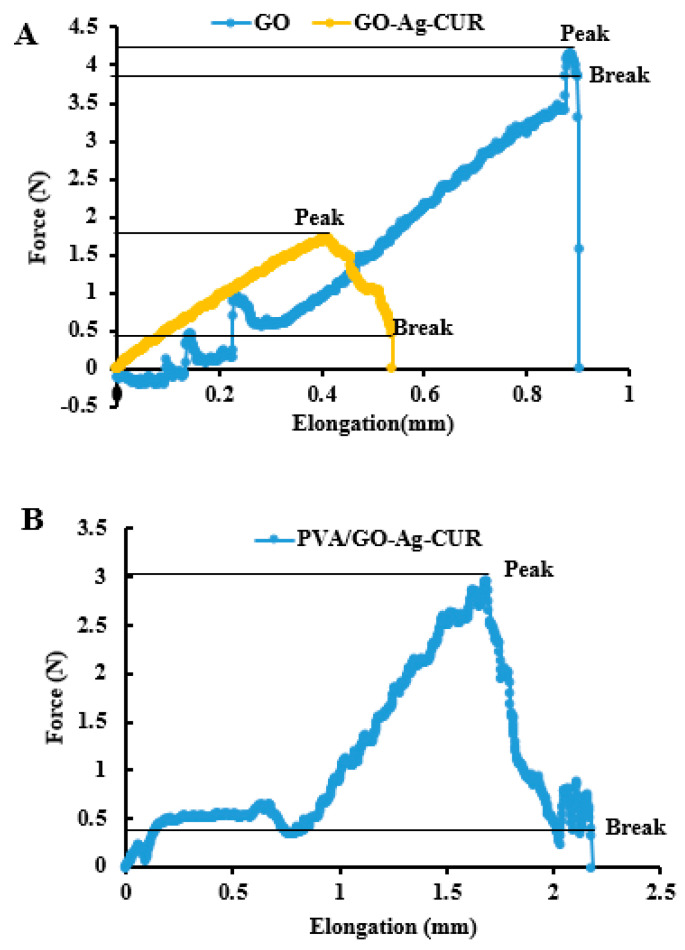
(**A**) The mechanical analysis of GO and GO-Ag-CUR nanostructure (**B**) PVA/GO-Ag-CUR nanofiber.

The mechanical analysis of GO, GO-Ag-CUR, and PVA/GO-Ag-CUR nanostructures is demonstrated in Figure 7A. Fracture strain and break point of GO-Ag-CUR exhibited an increase compared to GO alone. This could be due to the strong interaction between components in the GO-Ag-CUR nanostructure. Table 4 summarizes the Young’s modulus for GO, GO-Ag-CUR, and PVA/GO-Ag-CUR nanostructures. The presence of Ag and CUR beside GO in the GO-Ag-CUR nanostructure resulted in the improvement in tensile stress and the decrease in the Young’s modulus compared to those of GO. Figure 7B represents the mechanical analysis of PVA/GO-Ag-CUR nanofiber. According to Figure 7B, the incorporation of GO-Ag-CUR nanostructure into PVA hydrogel improved its mechanical attributes, such as peak and break point, tensile stress, fracture strain, and Young’s modulus, compared to those of the GO-Ag-CUR nanostructure. PVA chemical interaction with components present in the nanofiber might be responsible for this increase, validated by the FTIR analysis. The Young’s modulus for skin was measured in the literature to be between 2.9 and 150 MPa [75]. As a result, the fabricated nanofibers must fit this range in order to be considered as an ideal wound dressing. Considering findings from Table 4, we can conclude that the synthesized nanostructures actually have Young’s modulus comparable to that of skin.

### 3.6. Antibacterial Properties

We opted for the MIC method in order to evaluate the antibacterial activity of GO, GO-Ag and GO-Ag-CUR nanostructures against two *E. coli* (Gram-negative) and *S. aureus* (Gram-positive) bacteria. In this experiment, tetracycline was considered as a control group. The results are shown in Table 5. The replication of each test was three. The MIC of GO and GO-Ag against *E. coli* bacteria was measured to be 1.34 and 1.21 mg/mL, respectively. The MIC of GO and GO-Ag nanostructures against *S. aureus* bacteria was 2.13 and 1.46 mg/mL, respectively. According to the mentioned values, the addition of Ag nanoparticles to GO caused a reduction in MIC concentration and an improvement in the antibacterial property. These findings correlate fairly well with that of Cobos et al. [76]. Furthermore, the MIC of GO-Ag-CUR nanostructure against both bacteria was lower than that of GO and GO-Ag nanostructures, corroborating the excellent antibacterial property of the GO-Ag-CUR nanostructure. Cobos et al. [76] synthesized GO-Ag nanohybrids as nanofillers in cancer therapy. The MIC values for GO against both *E. coli* and *S. aureus* bacteria was 128 µg/mL. MIC concentration of GO-Ag against *E. coli* and *S. aureus* bacteria was 64 and 32 µg/mL, respectively. Zavareh et al. [77] prepared the copper (Cu^2+^)-bound GO adsorbent with antibacterial activity for eliminating aniline from water. MIC concentration of GO and GO-Cu against *E. coli* and *S. aureus* was measured to be 125 and 31.25 µg/mL, respectively, while the MIC of GO and GO-Cu adsorbents against *S. aureus* was 250 and 125 µg/mL, respectively.

### 3.7. Optical Density Measurement

**Figure 8:**
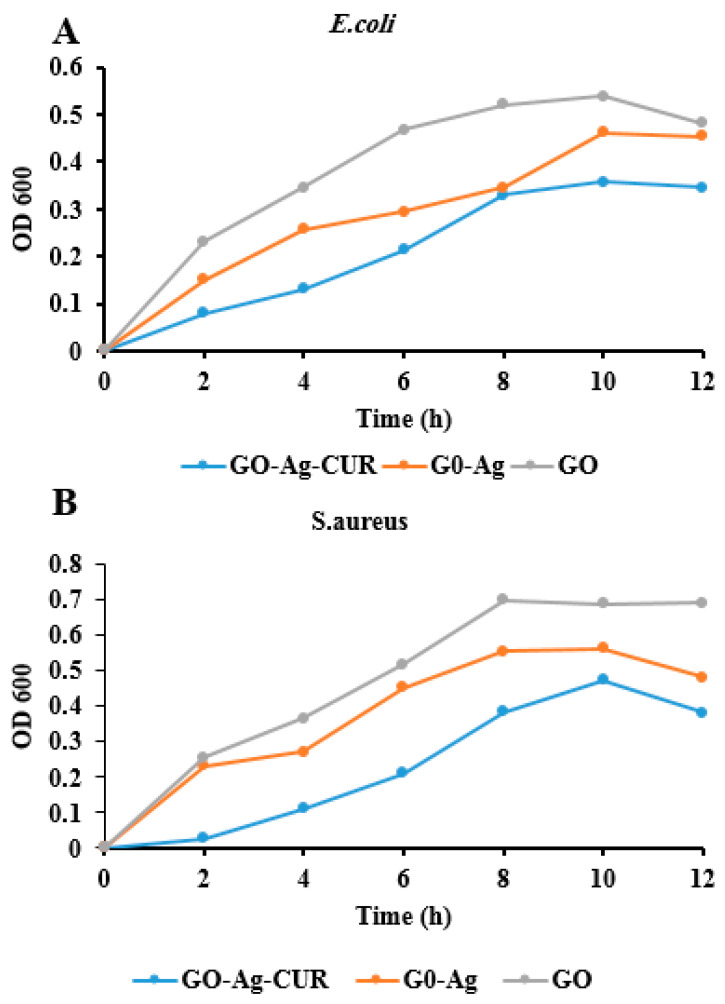
OD measurement of (**A**) *E.coli* and (**B**) *S. aureus* for MIC concentration of GO, GO-Ag, and GO-Ag-CUR.

For investigating the antibacterial activity of GO, GO-Ag and GO-Ag-CUR nanostructures against *E. coli* and *S. aureus* bacteria, we used a quantitative evaluation of the antibacterial activity based on the optical density (OD_600_). The growth curve of both *E. coli* and *S. aureus* bacteria in the presence of GO, GO-Ag and GO-Ag-CUR nanostructures is shown in Figure 8. This test was performed at MIC concentration for GO, GO-Ag and GO-Ag-CUR nanostructures over a 12-h period using 2-h intervals. As expected, GO-Ag-CUR inhibit the growth of both *E. coli* and *S. aureus* bacteria more than other nanostructures. This finding demonstrates the synergistic effect of GO, Ag, and CUR on the bacterial growth inhibition. The present results provide confidence for the MIC analysis.

### 3.8. CUR Loading and Encapsulation Efficiencies

When it comes to drug delivery system and wound dressing, loading and encapsulation efficiencies are of paramount significance, since poor loading capacity has a direct impact on the efficiency of a drug delivery platform or wound dressing [78]. Furthermore, as stated earlier, the major problem of CUR administration is its poor solubility and limited bioavailability [52]. Consequently, improving CUR loading and encapsulation efficiencies can be seen as big step forward for the application of CUR in wound dressing. To estimate loading and encapsulation efficiencies, equation 3 and 4 can be used. The loading efficiency was improved by the incorporation of Ag ions from 21 in the GO nanostructure to 56% in the GO-Ag nanostructure (Table 6). Fahimirad et al. [79] synthesized poly(ε-caprolactone) (PCL)-chitosan (CS)-CUR composite using the blending electrospinning technique for wound dressing with good antibacterial activity. Curcumin-loaded chitosan nanoparticles (CURCSNPs) were sprayed on the surface of the fabricated PCL-CS-CUR composite for improving the sustainable release of CUR at the wound site. CUR loading and encapsulation efficiencies of 42.6 and 74.2%, respectively, were found in the nanofibers. Regarding the delivery of CUR to animals’ wounds, Hamam et al. [80] prepared CUR-loaded mesoporous silica particles. The particles not only decrease inflammatory reactions by day 21 but also help the process of wound epithelization to be almost completed by day 21. Additionally, due to the high surface area and highly ordered arrangement of large pores in the structure of silica, the prepared particles showed high loading efficiency of 98% for CUR.

The encapsulation efficiency of CUR was estimated to be 60% in GO nanostructure and 85% in GO-Ag nanostructure, indicating the effective role of Ag nanoparticles in the improvement of encapsulation efficiency (Table 6). Kianvash et al. [81] prepared curcumin-propylene glycol nanoliposomes for skin burn therapy. To optimize the biocompatibility of the prepared nanoliposomes, they opted for a dose–response assessment. The optimized nanoliposomes showed encapsulation efficiency of 84.66%. Varaprasad et al. [82] successfully synthesized hydrogel silver nanocomposites containing CUR through a polymerization step in the presence of poly (vinyl sulfonic acid sodium salt) and then a trifunctional crosslinking step using the redox initiating system (ammonium persulphate/TMEDA). It was concluded that the incorporation of Ag nanoparticles in the prepared hydrogel increased encapsulation efficiency much more than Ag^+^ ions. They reported a maximum encapsulation efficiency of 87.9 related to a silver nanoparticle loaded hydrogel.

### 3.9. Release of CUR

**Figure 9:**
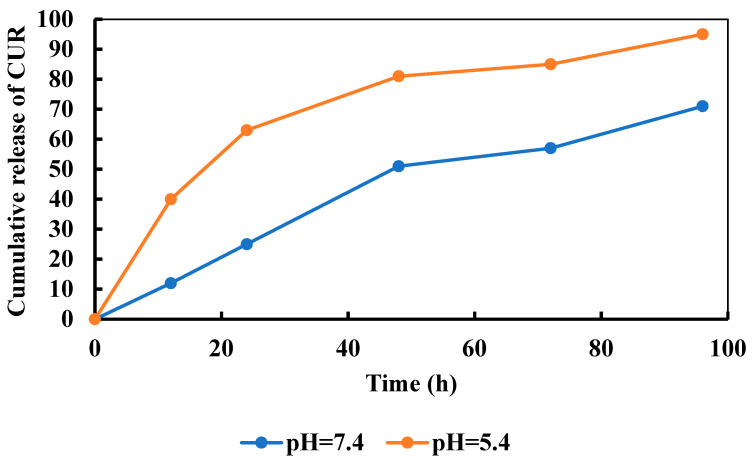
In vitro release profile of CUR from PVA/GO-Ag-CUR nanofiber in pH 5.4 and pH 7.4 at 37 °C at predetermined time intervals using the dialysis method.

To prove the pH-sensitivity of PVA/GO-Ag-CUR nanofibers, we studied the release mechanism. The dialysis technique was opted for evaluating the delivery of CUR from the nanofiber at two different pH of 7.4 and 5.4 at 37 °C (the typical human body’s temperature) for 96 h (Figure 9). After passing 24 h, the overall release of CUR was 25% at pH 7.4 from the PVA/GO-Ag-CUR nanofiber, while the cumulative release of CUR was 63% following 24 h at pH 5.4 (acidic medium). The release of CUR from PVA/GO-Ag-CUR nanofiber was 95% within 96 h in pH 5.4, whereas only 64% of the drug was released from the nanofiber within 96 h in pH 7.4. A decrease in the cumulative release of CUR at pH = 7.4 in comparison with pH = 5.4 may be ascribed to the interconnections between nanomaterials, polymer, and the drug, keeping the nanofiber integrity. The pH-sensitivity of the nanofibers is corroborated by the dissimilarity between the release mechanism at two pH of 5.4 and 7.4. In fact, the presence of protonated Ag^+^ groups and their interaction with other protonated components in acidic medium stimulates the repulsive force between the components of the nanofiber. Furthermore, the rapid swelling resulting from the penetration of buffer into disintegrated nanofiber increased the release rate while the components repelled each other [83].

### 3.10. Kinetics Modeling

The numerical modeling of the releasing process is critical. In designing a drug-loaded platform, these models are beneficial since they can predict mass transfer, drug delivery method, and pharmaceutical discharge kinetics of the nanostructure. Employing outcomes from the dialysis, the kinetics of the drug release was characterized. Zero-order, first-order, Higuchi, and Korsmeyer–Peppas models were used to model the discharge at pH 7.4 and 5.4. The kinetic modeling parameters and the models’ correlation coefficients for pH = 7.4 and pH = 5.4 are summarized in Table 7. According to the highest R^2^ values, Korsmeyer–Peppas and Higuchi models were found to cover the CUR release at pH 7.4 and 5.4, respectively. As already hypothesized, the first-order model was unsuitable due to the poor water-solubility of CUR. The Korsmeyer–Peppas model also showed a significant correlation constant for all release patterns, despite the fact that the Higuchi model exhibited a greater correlation constant at pH = 5.4. N values under 0.43 imply Fickian dispersion, and those over 0.85 represent case-II transfer in circular matrixes [84]. Table 7 shows that the n value for the PVA/GO-Ag-CUR nanofiber in pH 7.4 is 0.854, but this amount in pH 5.4 is only 0.398. Drug release in pH 5.4 seems to be well-controlled, according to these data [85].

### 3.11. MTT Assay

**Figure 10:**
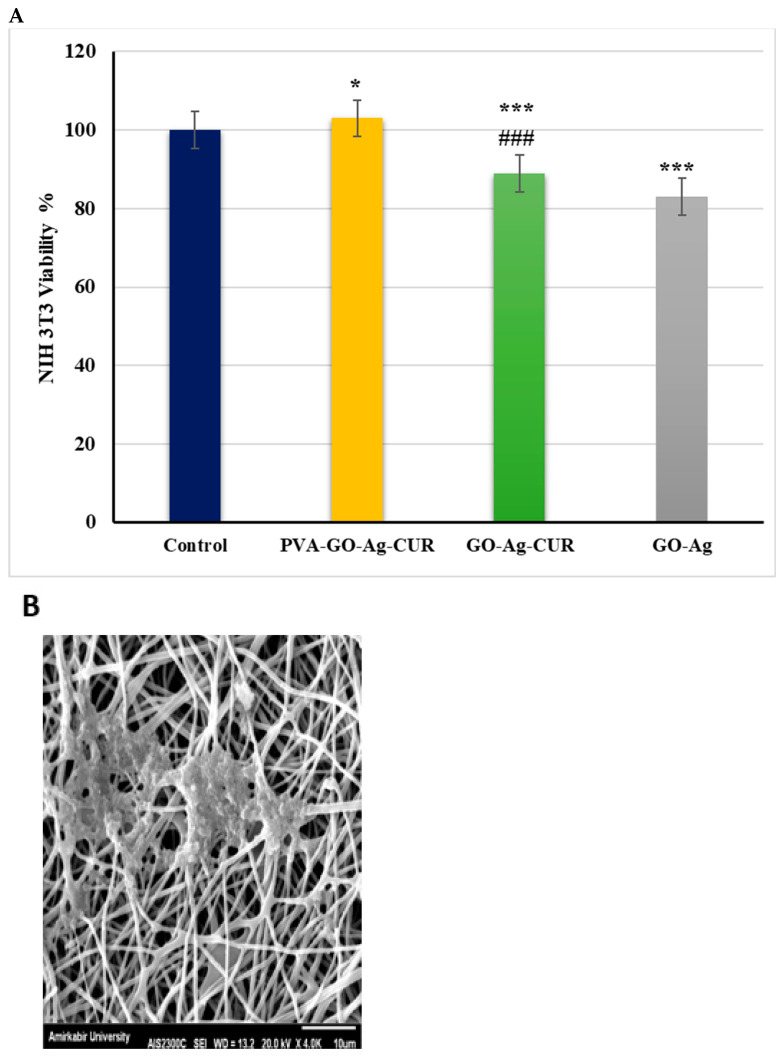
(**A**) The viability analysis of NIH 3T3 fibroblast cells treated with GO-Ag, GO-Ag-CUR, and PVA/GO-Ag-CUR groups after 24 h. Difference in viability between control and PVA-GO-Ag-CUR is significant with *p* < 0.005 (*). Difference between control and other groups is significant with *p* < 0.001 (***). Difference between PVA-GO-Ag-CUR and GO-AG-CUR is significant with *p* < 0.001 (###). (**B**) SEM image NIH 3T fibroblast cells cultured with PVA/GO-Ag-CUR nanofibers.

The cytotoxicity effect of GO-Ag, GO-Ag-CUR and PVA/GO-Ag-CUR nanostructures on NIH 3T3 fibroblast cells was determined after incubation for 24 h employing the MTT assay shown in Figure 10. We used NIH 3T3 fibroblast cells that received no treatment as control. Cell viability larger than 80% is considered for biocompatibility based on ISO standards [86]. As shown in Figure 10, all of the nanostructures are biocompatible because the resultant viability percentages are more than 80% in NIH 3T3 fibroblast cells. The viability of treated cells with PVA/GO-Ag-CUR is more than that of treated cells with GO-Ag and GO-Ag-CUR, confirming the effective role of PVA. The result indicates that PVA/GO-Ag-CUR showed not only no notable toxicity on NIH 3T3 fibroblast cells but also after one day improved the growth and multiplication of the fibroblast cells. All these findings led us to conclude the non-toxicity of PVA/GO-Ag-CUR nanofibers. The increased growth and proliferation of fibroblast cells by the presence of PVA/GO-Ag-CUR is in line with the results presented by Sayed et al. [87]. They concluded that PVA-silk fibroin nanofiber showed better growth and proliferation on HCFC fibroblast cells than the control group. In research carried out by Salmeh and co-workers [4], the effect of GO-arginine-Nigella sativa composite on the multiplication of NTH3T3 fibroblast cells was examined. They concluded that the rate of cell proliferation for the GO-arginine-Nigella sativa composite with a concentration of 2 µg/mL was about 127%, indicating non-toxicity and cell growth and proliferation effect of the fabricated composite.

## 4. Conclusions

In the present study, a wound dressing composed of PVA, GO, Ag, and CUR was fabricated to attain antibacterial properties, along with adequate biocompatibility and mechanical characteristics, using the electrospinning method. Using CCD and RSM, we found suitable processing conditions for the synthesis of PVA/GO-Ag-CUR nanostructures with suitable swelling behavior. The results of SEM, FTIR, and XRD confirmed the incorporation of all components into the fabricated nanostructures. The stability of nanostructures was revealed by zeta potential analysis. Homogeneous size distribution was obtained from DLS results. The incorporation of GO-Ag-CUR nanostructures into the PVA structure improved mechanical properties. Moreover, the prepared nanostructure showed not only antibacterial activities against *E. coli* and *S. aureus* bacteria but also positive roles in migration and proliferation of NIH 3T3 fibroblast cells. The release behavior of the fabricated nanofiber was pH-responsive, which results from the strong interactions between the components. Using Ag nanoparticles in the fabrication of the nanofibers resulted in much higher loading and encapsulation efficiencies. MTT assay showed that the viability of the fibroblast cells-treated PVA/GO-Ag-CUR nanofiber was higher in comparison with the control group, suggesting not only the good biocompatibility of the nanofiber but also the effectiveness of the nanofiber in cell adhesion, growth and proliferation. Consequently, it is hoped that our research will be helpful in solving problems associated with wound healing.

## Figures and Tables

**Table 1:** Matrix of CCD; factors and experimental responses.

Run	GO-Ag-CUR (mg/mL)	Volume Ratio of GA to GO-Ag-CUR (g/L)	Water-Uptake Capacity (%)
1	−1	−1	62 ± 3.2
2	+1	−1	78 ± 2.8
3	−1	+1	32 ± 2.2
4	+1	+1	53 ± 6.1
5	−1	0	43 ± 8.4
6	+1	0	56 ± 4.2
7	0	−1	59 ± 5.5
8	0	+1	37 ± 5.1
9	0	0	44 ± 4.7

**Table 2:** Analysis of variance.

Source	*p*-Value	F-Value
A	0.005	53.13
B	0.002	126.00
AB	0.438	0.80
A^2^	0.034	13.71
B^2^	0.060	8.68

**Table 3:** Analysis of variance after removing non-significant factors.

Source	*p*-Value	F-Value
A	0.002	55.97
B	0.000	132.74
A^2^	0.019	14.45
B^2^	0.039	9.14

**Table 4:** Young’s modulus for GO, GO-Ag-CUR, and PVA/GO-Ag-CUR nanostructures.

	GO	GO-Ag-CUR	PVA/GO-Ag-CUR
Young’s modulus (MPa)	83 ± 1.6	62 ± 4.7	106 ± 3.2

**Table 5:** MIC values of GO, GO-Ag, and GO-Ag-CUR nanostructures.

Bacteria	MIC			
	GO	GO-Ag	GO-Ag-CUR	Tetracycline
*S. aureus*	2.13 ± 3.6	1.46 ± 1.5	1.13 ± 7.6	0.9 ± 2.4
*E. coli*	1.34 ± 4.7	1.21 ± 2.3	0.96 ± 4.9	0.9 ± 3.6

**Table 6:** CUR loading and encapsulation efficiencies in GO and GO-Ag.

	GO	GO-Ag
Loading (%)	21 ± 6.9	56 ± 2.2
Encapsulation (%)	60 ± 1.7	85 ± 6.1

**Table 7:** The kinetic models’ parameters and correlation coefficients for estimating the release of CUR from PVA/GO-Ag-CUR nanofiber in pH 5.4 and pH 7.4.

		Zero-Order	First-Order	Higuchi	Korsmeyer–Peppas
pH = 5.4	R^2^	0.558	−0.187	0.959	0.948
		K0 = −1.212	K1 = −9.770	Kh = 9.374	K = 0.162 n = 0.398
pH = 7.4	R^2^	0.899	−0.349	0.928	0.975
		K0 = −0.771	K1 = −8.280	Kh = 15.19	K = 0.0173 n = 0.854

## Data Availability

Data are included within this article.

## Abbreviations

PVA	Polyvinyl alcohol
GO	Graphene oxide
Ag	Silver
CUR	Curcumin
GA	Glutaraldehyde
PBS	Phosphate buffer saline
DMSO	Dimethylsulfoxide
EDTA	Ethylenediaminetetraacetic acid
DMEM	Dulbecco’s Modified Eagle’s Medium
MIC	Minimum Initial Concentration
OD	Optical density
MTT	3-(4,5-Dimethyl-thiazol-2-yl)-2, 5-diphenyl-tetrazolium bromide
CCD	Central Composite Design
RSM	Response Surface Methodology
SEM	Scanning Electron Microscope
FESEM	Field Emission Scanning Electron Microscope
FTIR	Fourier Transform Infrared
DLS	Dynamic light scattering
XRD	X-ray diffraction
